# Associations of antidepressants and antipsychotics with lipid parameters: Do *CYP2C19*/*CYP2D6* genes play a role? A UK population-based study

**DOI:** 10.1177/02698811231152748

**Published:** 2023-02-11

**Authors:** Alvin Richards-Belle, Isabelle Austin-Zimmerman, Baihan Wang, Eirini Zartaloudi, Marius Cotic, Caitlin Gracie, Noushin Saadullah Khani, Yanisa Wannasuphoprasit, Marta Wronska, Yogita Dawda, David PJ Osborn, Elvira Bramon

**Affiliations:** 1 Mental Health Neuroscience Research Department, Division of Psychiatry, University College London, London, UK; 2 Social, Genetic and Developmental Psychiatry Centre, Institute of Psychiatry, Psychology and Neuroscience, King’s College London, London, UK; 3 Department of Pharmacy, Central and North West London NHS Foundation Trust, London, UK; 4 Epidemiology and Applied Clinical Research Department, Division of Psychiatry, University College London, London, UK; 5 Camden and Islington NHS Foundation Trust, London, UK

**Keywords:** Antidepressants, antipsychotics, lipids, cholesterol, pharmacogenetics, genetics

## Abstract

**Background:**

Dyslipidaemia is an important cardiovascular risk factor for people with severe mental illness, contributing to premature mortality. The link between antipsychotics and dyslipidaemia is well established, while evidence on antidepressants is mixed.

**Aims:**

To investigate if antidepressant/antipsychotic use was associated with lipid parameters in UK Biobank participants and if *CYP2C19* and *CYP2D6* genetic variation plays a role.

**Methods:**

Review of self-reported prescription medications identified participants taking antidepressants/antipsychotics. Total, low-, and high-density lipoprotein cholesterol (L/HDL-C) and triglycerides derived from blood samples. *CYP2C19* and *CYP2D6* metabolic phenotypes were assigned from genetic data. Linear regression investigated aims, adjusted for key covariates.

**Results:**

Of 469,739 participants, 36,043 took antidepressants (53% female, median age 58, 17% taking cholesterol-lowering medications) and 3255 took antipsychotics (58% female, median age 57, 27% taking cholesterol-lowering medications). Significant associations were found between use of each amitriptyline, fluoxetine, citalopram/escitalopram, sertraline, paroxetine and venlafaxine with higher total cholesterol, LDL-C, and triglycerides and lower HDL-C, compared to participants not taking each medication. Venlafaxine was associated with the worst lipid profile (total cholesterol, adjusted mean difference: 0.21 mmol/L, 95% confidence interval (CI): 0.17 to 0.26, *p* < 0.001). Antipsychotic use was significantly associated with lower HDL-C and higher triglycerides. In participants taking sertraline, *CYP2C19* intermediate metabolisers had higher HDL-C (0.05 mmol/L, 95% CI: 0.01 to 0.09, *p* = 0.007) and lower triglycerides (−0.17 mmol/L, 95% CI: −0.29 to −0.05, *p* = 0.007), compared to normal metabolisers.

**Conclusions:**

Antidepressants were significantly associated with adverse lipid profiles, potentially warranting baseline and regular monitoring. Further research should investigate the mechanistic pathways underlying the protective effects of the *CYP2C19* intermediate metaboliser phenotype on HDL-C and triglycerides in people taking sertraline.

## Introduction

Dyslipidaemia is an important cardiovascular risk factor (Expert Panel on Detection, Evaluation, and Treatment of High Blood Cholesterol in Adults, 2001) for people with severe mental illness (Osborn et al., 2015), contributing to premature mortality (Firth et al., 2019). The link between medications used in the treatment of psychosis with dyslipidaemia is well established (Li et al., 2020; Ribeiro et al., 2018; Rummel-Kluge et al., 2010). United Kingdom (UK) National Institute for Health and Care Excellence (NICE) guidelines recommend monitoring blood lipid profiles (i.e., total, low-, and high-density lipoprotein cholesterol (L/HDL-C), triglycerides and the total cholesterol to HDL-C ratio) in people prescribed such medications (National Institute for Health and Care Excellence, 2014). Evidence and guidance regarding medications used for depression, however, are mixed, with a paucity of high-quality studies (Hiles et al., 2016; Noordam et al., 2015; Raeder et al., 2006; Viscogliosi et al., 2020).

We use the terms ‘antipsychotic’ and ‘antidepressant’ to refer to medications in the ‘drugs for psychosis’ and ‘drugs for depression’ sections of the Neuroscience based Nomenclature (Nutt and Blier, 2016), respectively. Several important, evidence-based, interventions – including psychological therapies, antidepressant medications, exercise and non-invasive brain stimulation – are recommended in the treatment of depression (National Institute for Health and Care Excellence, 2022), with antidepressant medications widely prescribed. Several relatively small observational studies report associations between antidepressant use and dyslipidaemia (Noordam et al., 2015; Raeder et al., 2006), including higher triglycerides and lower HDL-C (Hiles et al., 2016; Viscogliosi et al., 2020), even though, in one study, most associations became non-statistically significant when adjusted for additional potential confounders (Viscogliosi et al., 2020). Some studies implicate tricyclics as most detrimental (Hiles et al., 2016), and others, selective serotonin reuptake inhibitors (Raeder et al., 2006). These studies, however, all had limited power to explore the relative effects of individual medications. A 2006 review concluded that certain antidepressants, such as tricyclics and mirtazapine, may negatively impact lipids more so than others (i.e., bupropion, venlafaxine, duloxetine), but noted low methodological quality of included studies and called for robust studies (McIntyre et al., 2006). The electronic medicines compendium, which provides access to manufacturers’ summaries of product characteristics for UK-licensed medicines, does not list lipid-related reactions for several antidepressants (e.g., amitriptyline, citalopram/escitalopram, fluoxetine), but lists increased cholesterol as common for paroxetine and venlafaxine and rare for sertraline (Electronic medicines compendium (EMC), 2022).

Antipsychotic medications are considered a mainstay of treatment in psychosis and multiple meta-analyses report links between individual antipsychotics and dyslipidaemia (Li et al., 2020; Ribeiro et al., 2018; Rummel-Kluge et al., 2010). A 2010 head-to-head meta-analysis of second-generation antipsychotics (48 blinded randomised trials) reported that olanzapine led to significantly greater increases in total cholesterol than aripiprazole, risperidone and ziprasidone and that quetiapine led to greater increases than risperidone (Rummel-Kluge et al., 2010). The electronic medicines compendium lists increased cholesterol and triglycerides as very common for quetiapine and olanzapine (the former also linked to adverse L/HDL-C); increased cholesterol as uncommon, and increased triglycerides rare, for risperidone; hypercholesterolemia and hypertriglyceridemia very rare for clozapine – while lipids are not mentioned for prochlorperazine, and lipid changes noted not clinically important for aripiprazole (Electronic medicines compendium (EMC), 2022).

Wide inter-individual variation in efficacy and adverse reactions of antidepressants and antipsychotics exists. Pharmacogenetics could play a significant role in individualising pharmacotherapy (Hicks et al., 2015, 2017). The Cytochrome P450 (CYP450) superfamily of enzymes are heavily involved in the metabolism of many prescribed medications (Lynch and Price, 2007); with *CYP2C19* and *CYP2D6* heavily involved for antidepressants and antipsychotics. The genes encoding these enzymes are highly polymorphic and thus represent promising pharmacogenetic targets (Hicks et al., 2015, 2017). Individuals can be phenotyped, respectively, based on *CYP2C19* and *CYP2D6* polymorphisms: ‘normal metabolisers’ carry two homozygous wild-type alleles and have normal enzymatic capacity; ‘poor metabolisers’ carry two loss-of-function alleles and have no enzymatic capacity; ‘intermediate metabolisers’ have reduced enzymatic capacity compared to normal metabolisers but greater capacity than poor metabolisers (e.g., one wild-type and one reduced capacity allele); and ‘rapid’ and ‘ultra-rapid metabolisers’ have greater than normal enzymatic capacity, due to either at least one increased function allele or duplications of functional allele(s) (Hicks et al., 2015, 2017). Phenotypes, of which distributions vary across ancestries, impact medication plasma concentrations and risk of adverse reactions (Austin-Zimmerman et al., 2021; Milosavljević et al., 2021), with poor metabolisers predicted as most at risk due to greater concentrations.

Few studies have investigated variation in *CYP2C19* and *CYP2D6* and lipid parameters in the context of antidepressants and antipsychotics. One study genotyped 150 inpatients with depression (most receiving antidepressants and over half also receiving antipsychotics) for *CYP2C9*, *CYP2C19* and *CYP2D6* and calculated four combinatory-gene indices, all of which significantly correlated with total cholesterol, LDL-C and HDL-C, but not triglycerides (Ruaño et al., 2011). A study of 76 patients taking risperidone found a significant negative change in HDL-C from pre-treatment to 8 weeks post-treatment in carriers of CYP2D6*2 and CYP2D6*65 (Lu et al., 2021). These studies, however, did not account for use of other medications such as statins, a mainstay of dyslipidaemia prevention and treatment, and were too small to draw conclusions.

### Aims

Given widespread and increasing use of antidepressants and antipsychotics (NHS Business Services Authority, 2022), it is important to study potential adverse effects, and their determinants, to inform optimal prescribing strategies and reduce risks at the individual- and population-level. Our aims were to investigate (1) if antidepressant/antipsychotic use was associated with lipid parameters in a large sample of participants from UK Biobank and (2) if *CYP2C19* and *CYP2D6* genetic variation plays a role influencing lipid parameters in participants taking antidepressants/antipsychotics. We hypothesised that (1) antipsychotics would be associated with worse lipid profiles than antidepressants and (2) the presence of one or more low function *CYP2C19* or *CYP2D6* alleles would be associated with increased risk of adverse lipid profiles.

## Materials and methods

### Study design

This population-based, observational, cohort study used genetic and cross-sectional data from UK Biobank (Bycroft et al., 2018; UK Biobank, 2007) – a major biomedical database with around 500,000 participants. UK Biobank received ethical approval from the North West – Haydock Research Ethics Committee (reference: 21/NW/0157). All participants provided written informed consent.

### Participants

UK Biobank methods have been described elsewhere (UK Biobank, 2007). We used data from the baseline visit, where, in brief, participants, aged 37–73, attended one of 22 UK assessment centres between 2006 and 2010 and completed an extensive set of measures, including questionnaires and interviews (e.g., demographics, medical history, medication use), and provided biological samples.

### Outcomes

Lipid parameters investigated were total cholesterol, LDL-C, HDL-C and triglycerides, measured in millimoles per litre (mmol/L), extracted directly from UK Biobank (originally derived from non-fasting venous blood samples analysed using a Beckman Coulter AU5800). To aid interpretation, we calculated and used the total cholesterol to HDL-C (TC:HDL) ratio as an additional outcome in analyses addressing aim one.

### Exposures

Exposures were (a) antidepressants/antipsychotics and (b) *CYP2C19* and *CYP2D6* genetic metabolic phenotypes. We reviewed self-reported ‘regular prescription medications’ data to identify all antidepressants/antipsychotics, considering both generic and proprietary names (identified through multiple sources (European Medicines Agency, 2022; The National Institute for Health and Care Excellence, 2022; U.S. Food and Drug Administration, 2022)), and combined equivalent medications under the generic name for analyses. We combined citalopram and escitalopram (the active enantiomer of citalopram) (The National Institute for Health and Care Excellence, 2022) as one medicine for analyses. We investigated aims in individual medications only if reported as being taken by ⩾1800 participants (consistent with a previous study) (Austin-Zimmerman et al., 2021). Medications not reaching this threshold were considered for inclusion in a higher-level combined group (e.g., all antipsychotics together).

For genetic exposures, we leveraged genome-wide genotyping and processing conducted centrally by UK Biobank (Bycroft et al., 2018). Genotyping was performed using the Affymetrix UK BiLEVE Axiom array on an initial sample (50,000 participants) and the Affymetrix UK Biobank Axiom® array (Affymetrix, Santa Clara, CA, USA) for all subsequent participants. These arrays include >820,000 variants (with good coverage of pharmacogenetics variants), with subsequent imputation of >90 million variants. Using the fully-imputed dataset, we then performed local quality control and assigned CYP450 metabolic phenotypes, as described previously (Austin-Zimmerman et al., 2021). In brief, to include and account for participants of non-European ancestry (European ancestry was determined centrally), two rounds of principal component analysis were conducted (using PC-AiR (Conomos et al., 2015) and PC-Relate (Conomos et al., 2016)), identifying four ancestry groups (East Asian, South Asian, African, admixed with predominantly European origin); participants not clustering with any main group were excluded. Subsequent processing excluded variants with minor allele frequency <1% and/or Fisher information score of <0.3 in each ancestry group; one of each pair of participants with a kinship score >0.083 (approximately third-degree relatives); and participants with >10% missingness, excessive genetic relatedness (>10 third-degree relatives); or a mismatch between self-reported and genetically-inferred sex.

To assign CYP metabolic phenotypes, we extracted *CYP2C19* and *CYP2D6* regions of interest (defined as one mega-base upstream of the 5′ end and one mega-base downstream of the 3′ end of the gene). Using an input map and reference panel from the 1000 genomes project (Delaneau et al., 2014), haplotypes were constructed based on genetic data, imputed using Beagle (version 5.0) (Browning et al., 2018), according to the star allele nomenclature system (PharmVar: Pharmacogene Variation Consortium, 2022) in line with Clinical Pharmacogenetics Implementation Consortium (CPIC) guidelines (Clinical Pharmacogenetics Implementation Consortium (CPIC), 2021; Hicks et al., 2015, 2017). Haplotypes containing no star allele-defining single-nucleotide polymorphism variants were classified as wild-type (*1) alleles for the corresponding gene. We grouped individuals into *CYP2C19* metabolic phenotypes based on activity of the individual haplotypes and resulting diplotypes (PharmVar: Pharmacogene Variation Consortium, 2022), and into *CYP2D6* phenotypes according to the activity score method (Gaedigk et al., 2008). We did not have data on *CYP2D6* copy number variants and were unable to define *CYP2D6* ultra-rapid metabolisers, or other whole gene deletions (e.g., CYP2D6*5). Of *CYP2D6* star alleles not called, *5 and *6 are most noteworthy; both are associated with poor metabolism, but each have a <3% frequency amongst Europeans (Zhou et al., 2017).

### Statistical analysis

Aim one analyses considered all participants with data on at least one lipid parameter and, from these, aim two considered participants with high-quality genetic data that reported taking antidepressants/antipsychotics.

In addition to lipid parameters, across each medication group – we described participants’ age at recruitment (years), sex, self-reported ethnic background, body mass index (BMI) (kg/m^2^), selected self-reported illnesses (depression, anxiety, schizophrenia, bipolar disorder) and concomitant use of cholesterol-lowering medications. We reported proportions for each lipid parameter using national categories (National Health Service, 2019). We included a comparison group of participants not taking medications of interest. In participants with high-quality genetic data that reported taking antidepressants/antipsychotics, we reported distributions of genetically-determined ancestry group, *CYP2C19* and *CYP2D6* metabolic phenotypes and use of strong/moderate inhibitors (as per United States Food and Drug Administration) (Center for Drug Evaluation and Research, 2021). Means with standard deviations, medians with interquartile ranges and/or counts and proportions were used, as appropriate.

We ran two linear regression models for each lipid parameter as a continuous outcome. The first, using all participants, investigated associations of use of each medication (main predictor) with each lipid parameter (outcome), adjusted for age (continuous), sex (binary) and use of cholesterol-lowering medication (binary). The second investigated the pharmacogenetic associations of *CYP2C19* and/or *CYP2D6* metabolic phenotype (main predictor(s)) with each lipid parameter (outcome). These models included participants with high-quality genetic data and were run in each medication group. *CYP2C19* and *CYP2D6* were modelled together where both genes are majorly involved in the metabolism of the medication (as per CPIC) (Clinical Pharmacogenetics Implementation Consortium (CPIC), 2021; Hicks et al., 2015, 2017), with normal metaboliser phenotypes used as reference. In addition to age, sex and cholesterol-lowering medication, these models were adjusted for genetic ancestry group (categorical) and, as relevant, use of strong/moderate *CYP2C19* or *CYP2D6* inhibitors (binary). We adjusted for strong/moderate inhibitors (e.g., fluoxetine and fluvoxamine for *CYP2C19* and paroxetine and quinidine for *CYP2D6*), as they play a major role in phenoconversion, a phenomenon whereby drug–gene or drug–drug–gene interactions may result in an observed phenotype different from the genetically-predicted phenotype (Cicali et al., 2021). We did not adjust for weak inhibitors as they have a much less clinically relevant impact on phenoconversion (Center for Drug Evaluation and Research, 2021) and are therefore not typically included when accounting for inhibitors (Cicali et al., 2021). We did not adjust for BMI in order to avoid the risk of overadjustment bias (Perry and Singh, 2018).

Effect estimates are reported with 95% confidence intervals (CIs) and uncorrected *p* values in tables and forest plots. Given analysis of three independent (LDL-C, HDL-C and triglycerides), and two highly correlated/combinatory (total cholesterol and TC:HDL ratio (the latter not included in pharmacogenetic analyses)) outcomes, we corrected for multiple testing by using an adjusted significance threshold of <0.013 (i.e., 0.05/4). Analyses were conducted in Stata/MP version 17.0 (StataCorp LLC, College Station, TX, USA).

## Results

### Participants

**Figure 1.**
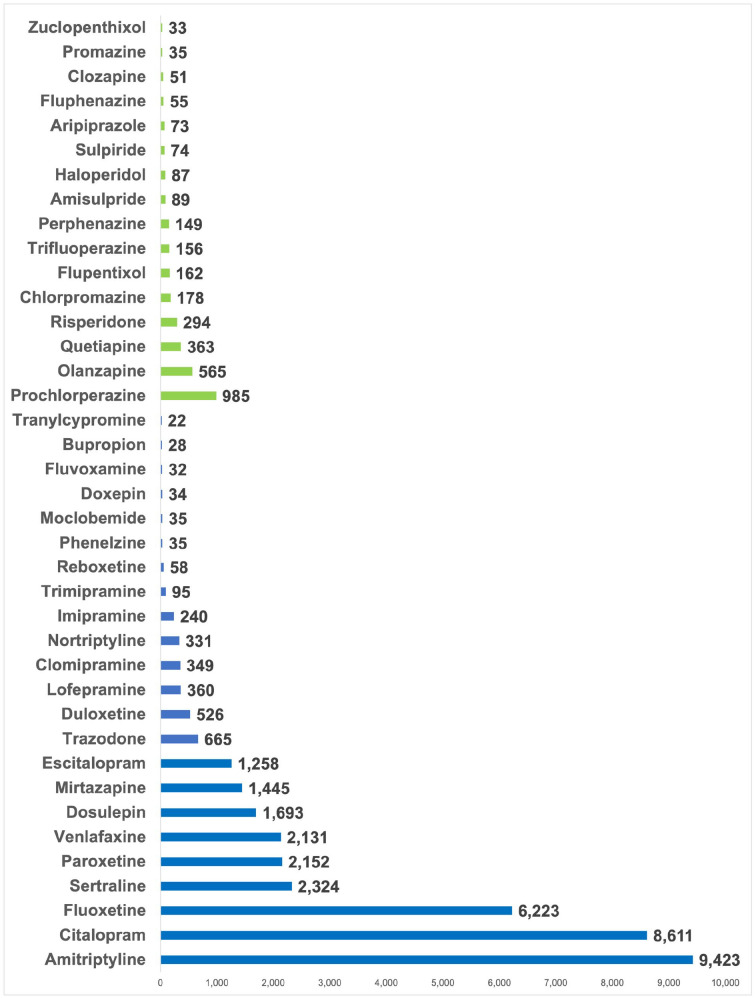
Frequency of antipsychotic and antidepressant medication use in UK Biobank participants. Numbers refer to the number of participants taking each medication. Antidepressants are shown in purple and antipsychotics in green. Medications were self-reported and are included if reported by at least 20 participants.

Overall, 469,739 participants had data for at least one lipid parameter. Of these, 36,043 reported taking at least one antidepressant, 3255 at least one antipsychotic, while 431,853 did not take either. Use of both an antidepressant and an antipsychotic was reported by 1412 participants. A wide range of medications were reported – with amitriptyline, citalopram and fluoxetine the most common antidepressants and prochlorperazine, olanzapine and quetiapine the most common antipsychotics (Figure 1). Amitriptyline, fluoxetine, citalopram/escitalopram, paroxetine, sertraline and venlafaxine met sample size threshold for individual medication analyses. No individual antipsychotic met threshold; all reported antipsychotics were therefore analysed together. Of participants taking antidepressants/antipsychotics, 33,472 had high-quality genetic data.

Sample characteristics, including demographics, unadjusted lipid parameters and genetic metabolic phenotypes, are shown in Table 1 and Supplementary Table 1. Compared to participants not taking antidepressants/antipsychotics, median age was similar across medication groups, but the proportion of females was consistently higher. Median BMI was highest in participants taking venlafaxine (28.4 kg/m^2^) and antipsychotics (28.2 kg/m^2^). Across antidepressants, participants taking amitriptyline had the lowest proportion of self-reported depression (17.5%) and anxiety (4.6%), while 27.9% taking antipsychotics self-reported schizophrenia or bipolar disorder. Nearly a quarter (24.7%) of participants taking antidepressants/antipsychotics were also taking cholesterol-lowering medications, compared to around a sixth (16.8%) in those not taking antidepressants/antipsychotics. Unadjusted lipid parameters stratified by cholesterol-lowering medication status are shown in Supplementary Table 2. For *CYP2C19*, most were either normal (13,939, 38.0%) or intermediate (10,860, 29.6%) metabolisers, with 1255 (3.4%) poor, 9069 (24.7%) rapid and 1535 (4.2%) ultra-rapid metabolisers. For *CYP2D6*, most (26,154, 71.4%) participants were normal metabolisers, with 1919 (5.2%) and 8585 (23.4%) poor and intermediate metabolisers, respectively.

**Table 1.** Sample characteristics.*.

Characteristic	No antidepressant/antipsychotic	Amitriptyline	Fluoxetine	Citalopram/escitalopram	Sertraline	Paroxetine	Venlafaxine	Any antipsychotic
*N*	431,853	9423	6223	9837	2324	2152	2131	3255
Age (years), median (IQR)	58 (50, 63)	60 (53, 64)	55 (48, 61)	56 (49, 61)	56 (49, 62)	58 (52, 63)	56 (49, 62)	57 (50, 63)
Sex – Female, *n* (%)	228,911 (53.0)	6653 (70.6)	4499 (72.3)	6800 (69.1)	1551 (66.7)	1410 (65.5)	1376 (64.6)	1886 (57.9)
Ethnic background, *n* (%)								
Asian	8647 (2.0)	130 (1.4)	46 (0.7)	135 (1.4)	24 (1.0)	21 (1.0)	14 (0.7)	74 (2.3)
Black	6966 (1.6)	102 (1.1)	46 (0.7)	61 (0.6)	11 (0.5)	14 (0.7)	15 (0.7)	83 (2.6)
Mixed	2490 (0.6)	60 (0.6)	41 (0.7)	71 (0.7)	20 (0.9)	7 (0.3)	8 (0.4)	38 (1.2)
White	406,403 (94.1)	9008 (95.6)	6024 (96.8)	9454 (96.1)	2245 (96.6)	2090 (97.1)	2056 (96.5)	2993 (92.0)
Other	5308 (1.2)	84 (0.9)	44 (0.7)	73 (7)	19 (0.8)	7 (0.3)	21 (1.0)	9 (1.5)
BMI (kg/m^2^), median (IQR)	26.7 (24.1, 29.7)	28.0 (25.0, 31.9)	27.9 (24.9, 31.8)	27.7 (24.6, 31.6)	27.8 (24.8, 31.5)	28.1 (25.1, 31.8)	28.4 (25.4, 32.0)	28.2 (25.1, 32.1)
BMI ⩾ 30 (kg/m^2^), *n* (%)	101,005 (23.4)	3327 (35.3)	2152 (34.6)	3333 (33.9)	781 (33.6)	767 (35.6)	818 (38.4)	1208 (37.1)
Self-reported illnesses, *n* (%)								
Depression	7625 (1.8)	1651 (17.5)	4378 (70.4)	6480 (65.9)	1419 (61.1)	1327 (61.7)	1565 (73.4)	1031 (31.7)
Anxiety	2434 (0.6)	434 (4.6)	594 (9.5)	1457 (14.8)	221 (9.5)	446 (20.7)	213 (10.0)	274 (8.4)
Schizophrenia	74 (<0.1)	9 (0.1)	22 (1.0)	40 (0.4)	25 (1.1)	7 (0.3)	27 (1.3)	557 (17.1)
Bipolar disorder/mania/manic depression	483 (0.1)	43 (0.5)	83 (1.3)	135 (1.4)	47 (2.0)	50 (2.3)	89 (4.2)	466 (14.3)
Cholesterol-lowering medication use, *n* (%)	72,371 (16.8)	2704 (28.7)	1333 (21.4)	2137 (21.7)	564 (24.3)	580 (27.0)	507 (23.8)	885 (27.2)
Total cholesterol (mmol/L), mean (SD)	5.69 (1.14)	5.68 (1.26)	5.81 (1.21)	5.70 (1.17)	5.78 (1.23)	5.79 (1.22)	5.83 (1.22)	5.55 (1.25)
Total cholesterol > 5 (mmol/L), *n* (%)	312,180 (72.3)	6547 (69.5)	4588 (73.7)	7090 (72.1)	1681 (72.3)	1580 (73.4)	1611 (75.6)	2151 (66.1)
LDL-C (mmol/L), mean (SD)	3.56 (0.87)	3.54 (0.96)	3.63 (0.92)	3.56 (0.89)	3.63 (0.93)	3.62 (0.92)	3.66 (0.94)	3.47 (0.94)
LDL-C > 3 (mmol/L), *n* (%)	314,330 (72.8)	6525 (69.3)	4562 (73.3)	7051 (71.7)	1697 (73.0)	1574 (73.1)	1589 (74.6)	2185 (67.1)
HDL-C (mmol/L), mean (SD)	1.45 (0.38)	1.41 (0.38)	1.45 (0.39)	1.44 (0.38)	1.41 (0.38)	1.42 (0.37)	1.41 (0.39)	1.35 (0.37)
HDL-C < 1 (mmol/L), *n* (%)	37,706 (8.7)	1089 (11.6)	577 (9.3)	984 (10.0)	272 (11.7)	219 (10.2)	237 (11.1)	481 (14.8)
Triglycerides (mmol/L), mean (SD)	1.73 (1.01)	2.00 (1.15)	1.91 (1.14)	1.88 (1.13)	1.98 (1.15)	2.06 (1.23)	2.07 (1.24)	2.05 (1.26)
Triglycerides > 2.3 (mmol/L), *n* (%)	89,533 (20.7)	2759 (29.3)	1655 (26.6)	2460 (25.0)	673 (29.0)	681 (31.6)	665 (31.2)	994 (30.5)
TC:HDL ratio, mean (SD)	4.12 (1.12)	4.23 (1.20)	4.20 (1.18)	4.18 (1.17)	4.32 (1.23)	4.28 (1.19)	4.37 (1.24)	4.33 (1.27)
*N* with genetic data passing quality control	–	8308	5510	8705	1981	1935	1906	2781
CYP2D6 metabolic phenotype,^1^ *n* (%)								
Normal metaboliser	–	5916 (71.2)	3928 (71.3)	6228 (71.6)	–	1368 (70.7)	1368 (71.8)	1958 (70.4)
Intermediate metaboliser	–	1961 (23.6)	1278 (23.2)	2007 (23.1)	–	457 (23.6)	433 (22.7)	682 (24.5)
Poor metaboliser	–	431 (5.2)	304 (5.5)	470 (5.4)	–	110 (5.7)	105 (5.5)	141 (5.1)
Strong/moderate CYP2D6 inhibitor(s) use, *n* (%)	–	342 (4.1)	5510 (100)^2^	89 (1.0)	–	1935 (100)^2^	16 (0.8)	277 (10.0)
CYP2C19 metabolic phenotype,^1^ *n* (%)								
Ultra-rapid metaboliser	–	366 (4.4)	–	361 (4.2)	76 (3.8)	–	–	–
Rapid metaboliser	–	1993 (24.0)	–	2180 (25.0)	476 (24.0)	–	–	–
Normal metaboliser	–	3162 (38.1)	–	3338 (38.4)	767 (38.7)	–	–	–
Intermediate metaboliser	–	2519 (30.3)	–	2555 (29.4)	595 (30.0)	–	–	–
Poor metaboliser	–	268 (3.2)	–	271 (3.11)	67 (3.4)	–	–	–
Strong/moderate CYP2C19 inhibitor(s) use, *n* (%)	–	246 (3.0)	–	55 (0.6)	23 (1.2)	–	–	–

BMI: body mass index; HDL-C: high-density lipoprotein cholesterol; LDL-C: low-density lipoprotein cholesterol; mmol/L: millimoles per litre; TC:HDL: total cholesterol to high-density lipoprotein cholesterol; IQR: interquartile range; SD: standard deviation.

^1^Medications were defined as CYP2C19 and/or CYP2D6 substrates in accordance with the Clinical Pharmacogenetics Implementation Consortium (CPIC, 2021; Hicks et al., 2015, 2017).

^2^Fluoxetine and paroxetine are themselves defined as strong inhibitors for CYP2D6 (Center for Drug Evaluation and Research, 2021).

* Cells may not add to column totals owing to missing data, See Supplementary Table 1 for an extended version of this table, which includes additional rows and indicators of missing data.

### Antidepressants, antipsychotics and lipid parameters

**Figure 2.**
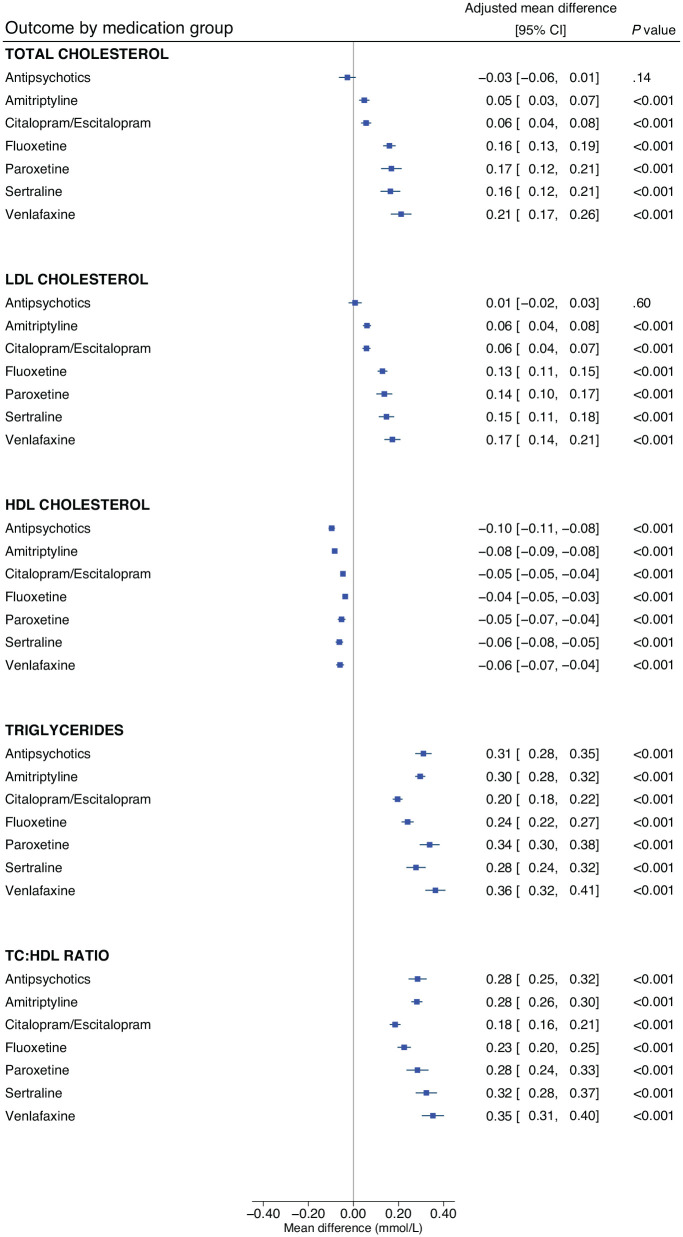
The associations of antidepressants and antipsychotics with lipid parameters. Linear regression models were adjusted for age, sex and concomitant use of cholesterol-lowering medications; effect estimates are coefficients for the main predictor variable, which was a binary variable defined by whether participants were taking the relevant medication (or not). A total of 469,591 participants contributed total cholesterol data, 468,708 for LDL cholesterol, 429,873 for HDL cholesterol and 469,216 for triglycerides. HDL: high-density lipoprotein; LDL: low-density lipoprotein; mmol/L: millimoles per litre; TC:HDL: total cholesterol to high-density lipoprotein cholesterol ratio.

Significant associations were found with the use of each antidepressant and each lipid parameter, respectively, when compared to participants not taking the medication (Figure 2, Supplementary Table 3). Antipsychotic use was significantly associated with lower HDL-C (mean difference: −0.10 mmol/L, 95% CI: −0.11 to −0.08, *p* < 0.001) and higher triglyceride levels (0.31 mmol/L, 95% CI: 0.28 to 0.35, *p* < 0.001), but not with total cholesterol or LDL-C.

Venlafaxine was associated with the highest levels of total cholesterol (mean difference: 0.21 mmol/L, 95% CI: 0.17 to 0.26, *p* < 0.001), followed by paroxetine (0.17 mmol/L, 95% CI: 0.12 to 0.21, *p* < 0.001) and sertraline (0.16 mmol/L, 95% CI: 0.12 to 0.21, *p* < 0.001). A similar pattern was observed for LDL-C. The lowest HDL-C levels were observed with antipsychotics and with amitriptyline (−0.08 mmol/L, 95% CI: −0.09 to −0.08, *p* < 0.001). The highest triglyceride levels were observed with venlafaxine (0.35 mmol/L, 95% CI: 0.31 to 0.40, *p* < 0.001) and sertraline (0.32 mmol/L, 95% CI: 0.28 to 0.37, *p* < 0.001). Results were similar when excluding participants taking any other antidepressants/antipsychotics from the reference group (Supplemental Table 4).

We conducted two post hoc analyses. As prochlorperazine was the most commonly reported antipsychotic, but is not typically currently used in the treatment of psychosis or bipolar disorder in the UK, we explored the impact of dropping participants taking (solely) prochlorperazine (*n* = 969) from analyses; results were consistent with the primary analyses (Supplementary Table 5). Given that amitriptyline is often prescribed for pain management, we conducted a secondary analysis considering amitriptyline only where participants also self-reported depression (*n* = 1651); results revealed a much larger adverse association of amitriptyline on lipid parameters in this subgroup (e.g., triglycerides: 0.42 mmol/L, 95% CI: 0.37 to 0.47, *p* < 0.001, TC:HDL ratio: 0.41, 95% CI: 0.36 to 0.47, *p* < 0.001), when compared the primary analyses (Supplementary Table 5). Characteristics of participants in the post hoc subgroups are compared with the overall group in Supplementary Table 6.

### The influence of CYP2C19 and CYP2D6 metabolic phenotypes

**Figure 3.**
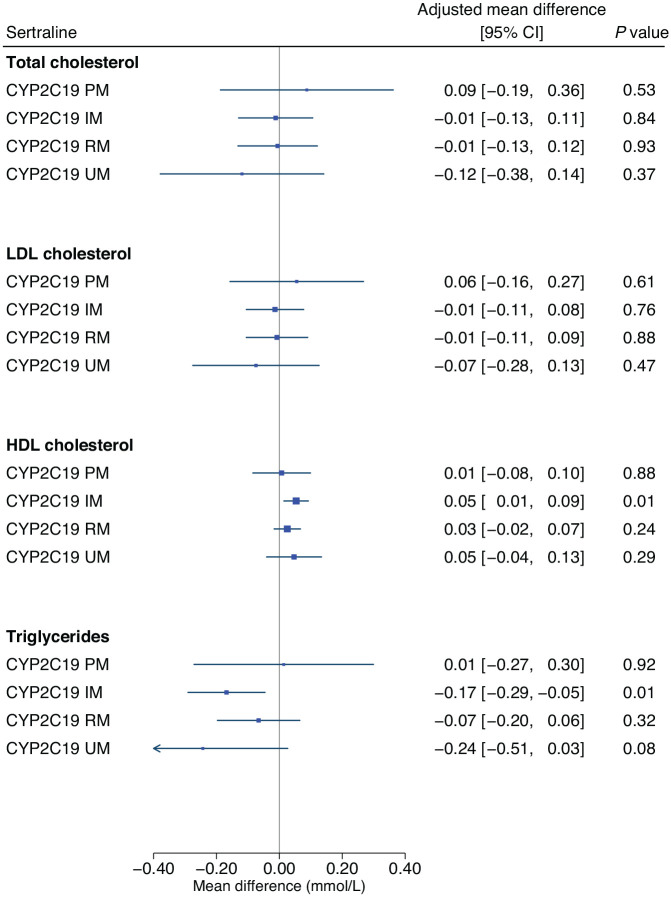
The influence of CYP2C19 metabolic phenotypes on lipid parameters in participants taking sertraline. Linear regression models were adjusted for age (continuous), sex (binary), genetically-determined ancestry group (categorical), concomitant use of cholesterol-lowering medications (binary) and use of strong/moderate CYP2C19 inhibitors (binary). The CYP2C19 normal metaboliser phenotype is the reference group. HDL: high-density lipoprotein; IM: intermediate metaboliser; LDL: low-density lipoprotein; mmol/L: millimoles per litre; PM: poor metaboliser; RM: rapid metaboliser; UM: ultra-rapid metaboliser.

Adjusted estimates of the influence of *CYP2C19* and *CYP2D6* metabolic phenotypes on lipid parameters across each medication group are shown in Table 2 and Supplementary Table 6, respectively. In participants taking sertraline, the *CYP2C19* intermediate metaboliser phenotype was significantly associated with an average 0.05 mmol/L higher HDL-C (95% CI: 0.01 to 0.09, *p* = 0.007) and with an average 0.17 mmol/L lower triglyceride level (95% CI: −0.29 to −0.05, *p* = 0.007), compared with normal metabolisers (Figure 3). As significant associations were in an unanticipated direction, we undertook post hoc analyses exploring the impact of cholesterol-lowering medications (Supplementary Table 8). Extension of the sertraline HDL-C model to include an interaction term for *CYP2C19* metabolic phenotype by cholesterol-lowering medications resulted in a main effect of the intermediate metaboliser phenotype larger than the primary analysis (0.08 mmol/L, 95% CI: 0.03 to 0.12, *p* = 0.001), with an intermediate metaboliser phenotype by cholesterol-lowering medications interaction effect in the opposite direction (−0.10 mmol/L, 95% CI: −0.19 to −0.01, *p* = 0.03, *n* = 126). Stratified analysis revealed a statistically strong effect of the *CYP2C19* intermediate metaboliser phenotype in participants not taking cholesterol-lowering medications (0.08 mmol/L, 95% CI: 0.03 to 0.12, *p* = 0.001, *n* = 424), but no evidence in those taking them. There was no evidence of an interaction in the extended triglycerides model, but some evidence in stratified analysis of a stronger association in participants not taking cholesterol-lowering medications (−0.15 mmol/L, 95% CI: −0.29 to −0.02, *p* = 0.03, *n* = 456).

**Table 2.** The influence of CYP2C19 metabolic phenotypes on lipid parameters in participants taking antidepressants or antipsychotics.

Predictors	Estimate (95% confidence interval) [*p* value]			
	Total cholesterol (mmol/L)	LDL cholesterol (mmol/L)	HDL cholesterol (mmol/L)	Triglycerides (mmol/L)
Amitriptyline				
CYP2C19 PM	−0.02 (−0.16, 0.12) [0.79]	−0.02 (−0.12, 0.09) [0.75]	0.03 (−0.011, 0.08) [0.17]	−0.10 (−0.25, 0.04) [0.16]
CYP2C19 IM	−0.02 (−0.08, 0.04) [0.49]	−0.02 (−0.06, 0.03) [0.49]	0.00 (−0.02, 0.02) [0.93]	−0.05 (−0.11, 0.01) [0.13]
CYP2C19 RM	−0.04 (−0.10, 0.02) [0.23]	−0.03 (−0.07, 0.02) [0.26]	0.00 (−0.03, 0.02) [0.66]	−0.01 (−0.07, 0.05) [0.78]
CYP2C19 UM	−0.06 (−0.17, 0.06) [0.36]	−0.03 (−0.12, 0.06) [0.48]	−0.04 (−0.08, −0.01) [0.025]	0.02 (−0.11, 0.14) [0.79]
Citalopram/escitalopram				
CYP2C19 PM	0.08 (−0.05, 0.21) [0.25]	0.05 (−0.05, 0.15) [0.35]	0.03 (−0.02, 0.08) [0.20]	0.03 (−0.10, 0.17) [0.65]
CYP2C19 IM	−0.02 (−0.07, 0.04) [0.54]	−0.02 (−0.06, 0.02) [0.42]	0.01 (−0.01, 0.02) [0.54]	−0.01 (−0.07, 0.04) [0.67]
CYP2C19 RM	−0.02 (−0.08, 0.04) [0.51]	−0.03 (−0.07, 0.02) [0.23]	0.02 (−0.00, 0.04) [0.08]	−0.05 (−0.11, 0.01) [0.10]
CYP2C19 UM	−0.05 (−0.17, 0.06) [0.38]	−0.04 (−0.13, 0.05) [0.37]	0.01 (−0.03, 0.05) [0.59]	−0.08 (−0.20, 0.04) [0.20]
Sertraline				
CYP2C19 PM	0.09 (−0.19, 0.36) [0.53]	0.06 (−0.16, 0.27) [0.61]	0.01 (−0.08, 0.01) [0.88]	0.01 (−0.27, 0.30) [0.92]
CYP2C19 IM	−0.01 (−0.13, 0.11) [0.85]	−0.01 (−0.11, 0.08) [0.76]	0.05 (0.01, 0.09) [0.007]	−0.17 (−0.29, −0.05) [0.007]
CYP2C19 RM	−0.01 (−0.13, 0.12) [0.93]	−0.01 (−0.11, 0.09) [0.88]	0.03 (−0.02, 0.07) [0.24]	−0.07 (−0.20, 0.06) [0.32]
CYP2C19 UM	−0.12 (−0.38, 0.14) [0.37]	−0.07 (−0.28, 0.13) [0.47]	0.05 (−0.04, 0.13) [0.29]	−0.24 (−0.51, 0.03) [0.08]

Linear regression models were adjusted for age (continuous), sex (binary), genetically-determined ancestry group (categorical), concomitant use of cholesterol-lowering medications (binary) and use of strong/moderate CYP2C19 or CYP2D6 inhibitors (binary). The CYP2C19 normal metaboliser phenotype is the reference group.

HDL: high-density lipoprotein; IM: intermediate metaboliser; LDL: low-density lipoprotein; mmol/L: millimoles per litre; PM: poor metaboliser; RM: rapid metaboliser; UM: ultra-rapid metaboliser.

No significant pharmacogenetic associations were found for other antidepressants or for antipsychotics. Use of strong/moderate *CYP2C19* inhibitors was not a significant predictor of any lipid parameter in CYP2C19 substrates. Use of strong/moderate *CYP2D6* inhibitors was associated with higher triglyceride levels in participants taking antipsychotics (0.29 mmol/L, 95% CI: 0.14 to 0.44, *p* = <0.001, *n* = 277). For venlafaxine, although *CYP2D6* inhibitor use was significant for total cholesterol and LDL-C, this subgroup was too small to meaningfully interpret (*n* = 16).

## Discussion

In this population-based, observational, cohort study using genetic and cross-sectional data on 469,739 participants from UK Biobank, we found that the use of amitriptyline, citalopram/escitalopram, fluoxetine, paroxetine, sertraline and venlafaxine were each all significantly associated with adverse levels of total cholesterol, LDL-C, HDL-C and triglycerides. In participants taking sertraline, we found that the *CYP2C19* intermediate metaboliser phenotype was significantly associated with higher HDL-C and lower triglycerides. Antipsychotic use was significantly associated with lower HDL-C and higher triglycerides.

Contrasting a previous review (McIntyre et al., 2006), venlafaxine was the antidepressant associated with the worst lipid profile in our study – with the highest levels of total cholesterol, LDL-C, triglycerides and greatest TC:HDL ratio. A similar profile was observed for paroxetine and sertraline, but, apart from HDL-C, the range of the CIs were generally less favourable for venlafaxine. Amitriptyline, in our primary analyses, was associated with more modest differences in total cholesterol and LDL-C but was associated with the lowest HDL-C levels. Citalopram/escitalopram appeared to have the least detrimental lipid profile – only fluoxetine was associated with a point estimate indicating less of a reduction in HDL-C (but with very similar CIs), though fluoxetine was associated with higher levels of the other lipids. Although amitriptyline was the most frequently reported antidepressant in our sample and associated with an adverse lipid profile, we anticipated a substantial proportion were taking amitriptyline for pain management (and therefore on lower doses than used for depression); in post hoc analyses that considered amitriptyline only where participants also reported depression, amitriptyline was associated with a much worse lipid profile, possibly reflecting a dose-response relationship, though this was based on a much smaller sample.

Our antidepressant results are consistent with some aspects of the 2021 Maudsley Prescribing Guidelines in Psychiatry (Taylor et al., 2021), but the latter highlight only venlafaxine, sertraline and mirtazapine (not studied here) as raising total cholesterol, and venlafaxine and mirtazapine as raising LDL-C. None are noted to impact HDL-C or triglycerides (notable given the largest effects in this study were observed for triglycerides, which contributes to metabolic syndrome). Overall, our results indicate that antidepressants are not benign with regards to lipid profiles – and more than typically assumed are in fact associated with adverse effects – highlighting the importance of studying individual medications. To date, a greater amount of efforts have been put into elucidating the cardiometabolic effects of antipsychotics, where baseline and annual monitoring is recommended in NICE guidelines (National Institute for Health and Care Excellence, 2014). Given that antidepressants are some of the most commonly prescribed medications – over 83 million were prescribed to over 8.3 million people in England alone in 2021/2022 (with numbers increasing year-on-year) (NHS Business Services Authority, 2022) – it is paramount to fully understand these effects and their determinants (genetic and environmental) in order to minimise adverse reactions for patients and to reduce cardiovascular risk at both the individual and population levels. Considering the magnitude of associations (and their associated CIs) identified, antidepressant choice may be most clinically relevant for patients at high risk for cardiovascular morbidity, including those with severe mental illness or pre-existing cardiovascular disease, where, for example, prescribing venlafaxine may be particularly detrimental to cardiovascular health and require a detailed risk-benefit analysis. Policymakers and guideline panels should consider whether the introduction of baseline and regular (e.g., annual) monitoring of lipids may be warranted, especially in high-risk groups prescribed antidepressants. Antidepressants in our study reflect contemporary prescribing in England (Statista, 2022), but further research into the effects of others (e.g., mirtazapine, duloxetine, trazadone) is needed.

Our pharmacogenetic results suggest that, in people taking sertraline, the *CYP2C19* intermediate metaboliser phenotype could be protective for HDL-C, possibly offsetting the overall lower HDL-C associated with sertraline and may also limit the higher triglyceride levels otherwise associated with sertraline. This is an important finding given that sertraline was dispensed over 20 million times in England in 2021 (Statista, 2022). As the *CYP2C19* intermediate metaboliser phenotype is relatively common (30% in this sample) and associated with clinically significant higher levels of sertraline exposure (Milosavljević et al., 2021), these results may have significance at the population-level, warranting replication and further investigation. *A priori*, we hypothesised that the presence of one or more low function *CYP2C19* or *CYP2D6* alleles would be associated with increased risk of adverse reactions such as an altered lipid profile. A similar paradoxical finding, of less adverse events in those with reduced *CYP2C19* metabolic activity taking sertraline, has also been reported in a paediatric sample (though lipids were not reported) (Rossow et al., 2020). These results suggest that reduced *CYP2C19* activity does not represent a general mechanism of increased risk of adverse reactions from *CYP2C19* substrates – instead, *CYP2C19* function may interact in complex (non-linear) ways with specific metabolic pathways to possible reactions, some of which may be beneficial. For sertraline, post hoc analyses identified that the effect of the *CYP2C19* intermediate metaboliser phenotype on HDL-C was observed in participants not taking cholesterol-lowering medications. Statins may therefore inhibit the observed protective effect through a much stronger impact on lipid metabolism in order to achieve their primary therapeutic target (LDL-C reduction). Given increasing polypharmacy, further research into possible interactions, both drug-drug and drug-drug-gene, on clinical outcomes and adverse reactions is warranted. Nevertheless, we did not find evidence for a role of *CYP2C19* or *CYP2D6* metabolic phenotypes on lipid parameters in other medications studied – research into other genes (e.g., *HTR2A*; Noordam et al., 2015; *NCAM1* and *KIAA1211*; Fjukstad et al., 2021) could be informative, as well as into other biological mechanisms to explain how antidepressants impact lipids.

We were surprised not to find an association between antipsychotic use and total cholesterol or LDL-C, given that this relationship is well established (Electronic medicines compendium (EMC), 2022; Li et al., 2020; National Institute for Health and Care Excellence, 2014; Ribeiro et al., 2018; Rummel-Kluge et al., 2010; Taylor et al., 2021). This could have been due to the population-based sampling used, which resulted in a relatively low number of participants taking each medication – no individual antipsychotic reached the ⩾1800-participant threshold. Our analyses therefore considered all antipsychotics as one potentially heterogeneous group – post hoc analyses; however, excluding the most frequently reported antipsychotic, prochlorperazine (commonly used for nausea) was consistent. It is also likely that many participants taking antipsychotics in our study were not taking them for psychosis or bipolar disorder, but rather for nausea, anxiety, hiccups, and therefore may have been on substantially lower doses. Another explanation could relate to clinical cardiovascular management. Almost 30% of participants taking antipsychotics also took cholesterol-lowering medications, nearly double than in the group not taking antipsychotics/antidepressants. Given known cardiometabolic adverse reactions, and the role of cardiovascular morbidity in the premature mortality in this population, UK general practitioners and psychiatrists have placed particular emphasis on managing cardiovascular risks and promoting health behaviours, including the prescription of statins and promotion of smoking cessation, a healthy diet and exercise. This area has also been the subject of UK government public health initiatives (Public Health England, 2018) and interventional research (Osborn et al., 2018). It is possible that closer monitoring could be limiting the detrimental effects of antipsychotics (noting participants were recruited from 2006 to 2010); different results may be observed in other settings.

This study has several limitations. Data on medication was self-reported and, along with lipids, measured at one time-point. Data on doses prescribed, medication plasma concentrations, duration of therapy and treatment indication would have enabled more sophisticated and detailed analyses. Future studies could consider other lipid parameters (e.g., very LDL-C), and randomised studies, controlling for other potential environmental confounders (e.g., diet, physical activity, tobacco, drug and alcohol use) and with multiple sampling time-points, are needed to provide more definitive evidence. It is also possible that certain psychiatric conditions or symptoms impact lipid parameters directly (Hiles et al., 2016; McIntyre et al., 2006) and/or interact with medications. Despite the large sample size, the number of non-normal metabolisers (particularly poor metabolisers) was much more modest – as expected, given the prevalence of polymorphisms that result in non-normal phenotypes, limiting statistical power and precision in our pharmacogenetic analyses. Future studies will need to employ different methods, such as oversampling, to ensure larger numbers of non-normal metabolisers. We were unable to define *CYP2D6* ultra-rapid metabolisers (any such individuals would be treated as normal metabolisers by default), but the prevalence of this phenotype is very rare (around 3% of Europeans; Gaedigk et al., 2017). Furthermore, we followed Pharmacogene Variation Consortium and CPIC guidelines to assign metabolic phenotypes from genetic data; these guidelines are generally similar to those from the Dutch Pharmacogenetics Working Group, but some important differences exist (e.g., the latter does not use the CYP2C19 rapid metaboliser phenotype) (Bank et al., 2018).

Around 94% of our sample were of white ethnicity and, when compared to the 2011 England and Wales population estimate (86%) (Office for National Statistics, 2015), this highlights that all other ethnicities were under-represented, potentially limiting generalisability, especially as cardiovascular risk is greater in some other ethnicities. It should also be noted that stigma and discrimination of both mental illness (Serafini et al., 2011) and taking psychiatric medications (Townsend et al., 2022) is prevalent and likely impacts service use, diagnosis, medication adherence as well as self-reports in this study.

This study also has strengths. We included a very large sample of participants from UK Biobank – enabling robust comparisons, particularly across individual antidepressants, comparing favourably to previous studies. Our pharmacogenetic analyses did not exclude participants of non-European ancestry, a practice that has been common in genetic studies (Ben-Eghan et al., 2020). Analyses were adjusted to account for key co-variates (e.g., cholesterol-lowering medications and *CYP2C19* /*CYP2D6* inhibitors). To our knowledge, this is also the first large study to investigate CYP450 metabolic phenotypes in this context.

## Conclusion

Commonly prescribed antidepressants were significantly associated with adverse lipid profiles – potentially warranting the introduction of baseline and regular monitoring of lipids, especially in high-risk groups prescribed them, in a similar way to existing recommendations for antipsychotics. Venlafaxine was associated with the worst lipid profile and might be avoided in those at high risk of cardiovascular morbidity, whereas citalopram/escitalopram had the smallest effect sizes for raised lipids and may be preferable in this group. Further research should investigate the mechanistic pathways underlying the protective effects of the *CYP2C19* intermediate metaboliser phenotype on HDL-C and triglycerides in people taking sertraline. Antipsychotic use was not associated with total cholesterol or LDL-C in our sample, possibly due to heterogeneity, modest statistical power and/or co-prescribed cholesterol-lowering medication, but was associated with lower HDL-C and higher triglycerides.

## Supplemental Material

sj-docx-1-jop-10.1177_02698811231152748 – Supplemental material for Associations of antidepressants and antipsychotics with lipid parameters: Do CYP2C19/CYP2D6 genes play a role? A UK population-based study Supplemental material, sj-docx-1-jop-10.1177_02698811231152748 for Associations of antidepressants and antipsychotics with lipid parameters: Do CYP2C19/CYP2D6 genes play a role? A UK population-based study by Alvin Richards-Belle, Isabelle Austin-Zimmerman, Baihan Wang, Eirini Zartaloudi, Marius Cotic, Caitlin Gracie, Noushin Saadullah Khani, Yanisa Wannasuphoprasit, Marta Wronska, Yogita Dawda, David PJ Osborn and Elvira Bramon in Journal of Psychopharmacology
